# Characterisation of recent foot-and-mouth disease viruses from African buffalo (*Syncerus caffer*) and cattle in Kenya is consistent with independent virus populations

**DOI:** 10.1186/s12917-015-0333-9

**Published:** 2015-02-03

**Authors:** Sabenzia Nabalayo Wekesa, Abraham Kiprotich Sangula, Graham J Belsham, Kirsten Tjornehoj, Vincent B Muwanika, Francis Gakuya, Dominic Mijele, Hans Redlef Siegismund

**Affiliations:** Foot-and-Mouth Disease Laboratory, Embakasi P. O. Box 18021, 00500 Nairobi, Kenya; Department of Environmental Management, College of Agricultural and Environmental Sciences, Makerere University, P. O. Box 7062/7298, Kampala, Uganda; National Veterinary Institute, Technical University of Denmark, Lindholm DK-4771 Kalvehave, Denmark; Kenya Wildlife Service, Veterinary Services Department, P.O Box 40241 (00100), Nairobi, Kenya; Department of Biology, University of Copenhagen, Ole Maaløes Vej 5, DK-2200 Copenhagen, Denmark

**Keywords:** African buffalo, Cattle, Control, Epidemiology, Foot-and-mouth disease, Lineages

## Abstract

**Background:**

Understanding the epidemiology of foot-and-mouth disease (FMD), including roles played by different hosts, is essential for improving disease control. The African buffalo (*Syncerus caffer*) is a reservoir for the SAT serotypes of FMD virus (FMDV). Large buffalo populations commonly intermingle with livestock in Kenya, yet earlier studies have focused on FMD in the domestic livestock, hence the contribution of buffalo to disease in livestock is largely unknown. This study analysed 47 epithelia collected from FMD outbreaks in Kenyan cattle between 2008 and 2012, and 102 probang and serum samples collected from buffalo in three different Kenyan ecosystems; Maasai-Mara (MME) (*n* = 40), Tsavo (TSE) (*n* = 33), and Meru (ME) (*n* = 29).

**Results:**

Antibodies against FMDV non-structural proteins were found in 65 of 102 (64%) sera from buffalo with 44/102 and 53/102 also having neutralising antibodies directed against FMDV SAT 1 and SAT 2, respectively. FMDV RNA was detected in 42% of the buffalo probang samples by RT-qPCR (Cycle Threshold (Ct) ≤32). Two buffalo probang samples were positive by VI and were identified as FMDV SAT 1 and SAT 2 by Ag-ELISA, while the latter assay detected serotypes O (1), A (20), SAT 1 (7) and SAT 2 (19) in the 47 cattle epithelia. VP1 coding sequences were generated for two buffalo and 21 cattle samples. Phylogenetic analyses revealed SAT 1 and SAT 2 virus lineages within buffalo that were distinct from those detected in cattle.

**Conclusions:**

We found that FMDV serotypes O, A, SAT 1 and SAT 2 were circulating among cattle in Kenya and cause disease, but only SAT 1 and SAT 2 viruses were successfully isolated from clinically normal buffalo. The buffalo isolates were genetically distinct from isolates obtained from cattle. Control efforts should focus primarily on reducing FMDV circulation among livestock and limiting interaction with buffalo. Comprehensive studies incorporating additional buffalo viruses are recommended.

**Electronic supplementary material:**

The online version of this article (doi:10.1186/s12917-015-0333-9) contains supplementary material, which is available to authorized users.

## Background

Numerous species of cloven-hoofed wildlife and livestock, including buffalo, impala, cattle, sheep, goats and pigs are affected by foot-and-mouth disease (FMD) [1]. The disease is clinically characterised by fever, lameness and vesicular lesions on the tongue, feet, snout/muzzle and teats of various susceptible species [2]. Globally, the disease causing agent, FMD virus (FMDV), exists in seven different serotypes (O, A, C, Asia 1, SAT 1, SAT 2 and SAT 3) and vaccination against (or infection with) one serotype does not cross-protect against other serotypes, hence the need for constant surveillance of circulating strains for appropriate vaccine selection [3].

In the eastern Africa region, including Kenya, four of the seven serotypes (O, A, SAT 1 and SAT 2) were previously known to circulate [4], but recently in 2013, SAT 3 was isolated from an apparently healthy long-horned Ankole calf in Uganda [5]. This multiplicity of serotypes, combined with the co-existence of a number of different wild and domestic hosts within large geographical areas, makes our understanding of the epidemiology and control of this disease complicated [6]. In eastern Africa, existing policies largely comprise vaccination and livestock movement control. Infection by the virus may result in substantial economic losses; these include production losses (e.g. reduced milk yields, lameness in draught animals, loss of weight, abortions, delayed conception, peri-natal mortality) as well as effects from restrictions on sales and exports of livestock and livestock products [7].

The severity of FMD varies from host to host, e.g. cattle commonly suffer acute, clinically apparant infections [2], while in the African buffalo (*Syncerus caffer*) the disease is usually subclinical [8,9] and hence is not easy to detect. It has been reported that within wildlife, the African buffalo are reservoirs for the SAT serotypes [8,10] and may play a role in the maintenance and spread of these serotypes to livestock [9] as reported in southern Africa. Moreover, this buffalo species is capable of harbouring the virus for as long as 5 years within an individual animal and for 24 years within a single herd [11]. Animals with these long-term infections are referred to as persistently infected animals or carriers, and are defined as animals in which the virus can be detected from the oesophago-pharyngeal scrapings (OP/probang sample) at 28 days or more after infection [12,13]. Unlike in southern Africa, the role of the African buffalo in the epidemiology of FMD is still unclear in eastern Africa [14,15], yet buffalo interact with livestock, grazing together in the vast and numerous un-fenced wildlife ecosystems.

It has been argued that wildlife might act as a source of sporadic disease occurrence in livestock with negative impacts on the harmonious co-existence of these species [16,17]. On the one hand, there is experimental evidence that FMD may spread from buffalo to cattle, while on the other hand, there is a lack of adequate supportive scientific evidence for the role of wildlife in the epidemiology of FMD in livestock [18]. Indeed, some studies have argued that FMD may be predominantly a disease of livestock [17,19] and that the spread of FMDV among livestock may be more associated with human activities than with wildlife [20]. Moreover, the importance of enhancing our understanding of disease spread at the wildlife-livestock interface and the need to balance biodiversity management with livestock production have been emphasized previously [16,21]. It is therefore necessary to study the disease spread at this interface to ensure that appropriate policies and control measures are implemented. This should help to protect the wildlife heritage and concurrently promote harmonious, profitable and sustainable livestock-wildlife interaction.

According to unpublished Kenya Wildlife Service (KWS) records, Kenya has an estimated total population of 26,325 buffalo distributed among numerous parks, reserves, sanctuaries and ranches found within several major ecosystems including Tsavo, Meru, Laikipia/Samburu, Amboseli, Nakuru and Maasai-Mara. This buffalo population and other less susceptible wildlife species complements the numerous domestic FMDV-susceptible hosts in Kenya, including 17.5 million cattle, 27.7 million goats, 17.1 million sheep and 300,000 domestic pigs recorded during the 2009 national animal census [22]. Records at the national Foot-and-Mouth Disease Laboratory (FMDL), Embakasi, show that previous studies on FMD in Kenya have been mainly focused on cattle and not other susceptible domestic species such as pigs [23] and only to a minor extent on wildlife. However, in 1979, a field survey isolated SAT 1 and SAT 2 FMDVs from buffalo populations in the southern part of Kenya [24], while a more recent study (1994-2002) established a higher seroprevalence of antibodies against FMDV in buffalo than in other susceptible wildlife species, but also highlighted some limitations of the specificity of the serological tests that were used [25].

Elsewhere in the east African region, studies of buffalo within neighbouring Uganda’s Queen Elizabeth National Park isolated SAT 3 virus in 1997 [26], reported antibodies against FMDV serotypes O, SAT 1, SAT 2 and SAT 3 in sera collected during 2001-2003 [15], and successfully isolated and genetically characterized SAT 1 and SAT 2 viruses in 2005-2008 [27].

This study, aimed at determining the presence of antibodies against different serotypes of FMDV within buffalo populations in selected wildlife ecosystems in Kenya and at comparing FMDV isolates from these buffalo populations with the FMDVs found in cattle within this country and elsewhere in Africa. The study endevoured to contribute towards generating reliable information regarding FMDV circulation in eastern Africa.

## Methods

### Ethical approval

This study was ethically approved by the Kenya Wildlife Services (permit no. KWS/BRM/5001) and undertaken in collaboration with the Department of Veterinary Services (DVS) of the Ministry of Livestock Development in Kenya under the Transboundary Animal Diseases in East Africa (TADEA) project, DFC no. 10-006KU.

### Samples from buffalo

**Figure 1:**
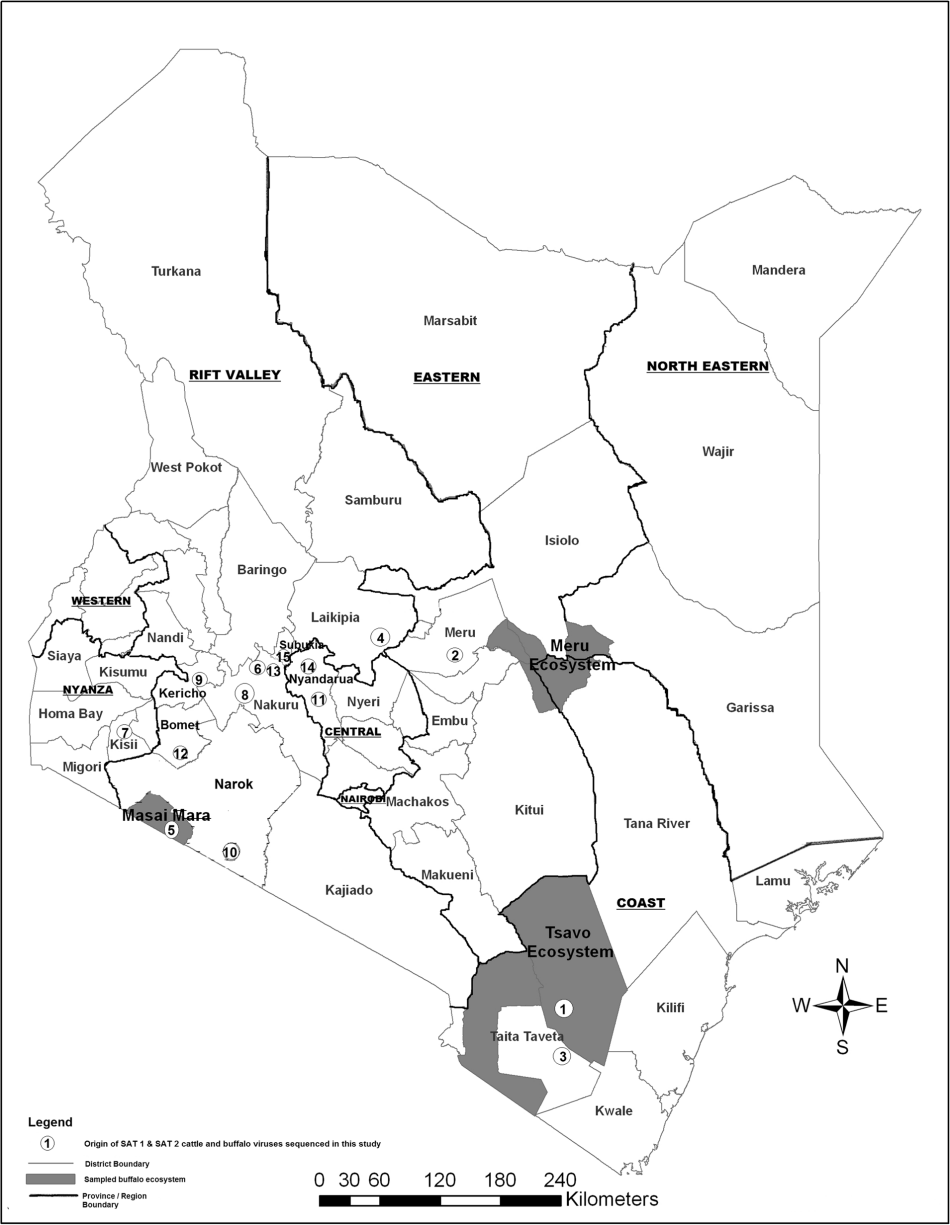
**Map of Kenya showing sampled wildlife ecosystems (shaded), administrative regions (underlined) and districts.** Circles with numbers indicate geographic origins of the 15 SAT 1 and SAT 2 foot-and-mouth disease viruses (FMDVs) isolated from buffalo and cattle sample analysed in this study. The numbers correspond to the serial numbers in Table 1. The map was created using ArcGIS (ArcMap v 9.3) copyright 2008 ESRI.

The buffalo sampling was carried out between March and July 2012. The study was designed as a cross-sectional study targeting buffalo populations interacting with domestic animals regardless of their age. The buffalo were clustered into three major buffalo ecosystems located within four out of the eight major administrative regions/provinces of Kenya (North-Eastern, Eastern, Rift Valley and Coast) (Figure 1). These include the Meru ecosystem (ME), represented by Meru National Park, the Maasai-Mara ecosystem (MME), represented by Maasai-Mara National Reserve and the Tsavo ecosystem (TSE), represented by Tsavo East National Park. These ecosystems have estimated populations of 4069, 3030 and 7281 buffalo, respectively, according to the KWS records. Within these ecosystems, there is a high level of interaction between livestock and wildlife. MME and TSE have open savannah-type vegetation, which eases the capture and handling of the animals, while ME has patches of wooded grassland vegetation.

Two groups of veterinarians and technicians in two separate vehicles carried out the buffalo sampling; one identified the herds and chemically immobilized the animals as described by [28], while the other traced, marked and sampled the immobilized animals, taking records of geographical location, age by dentition [29], sex, clinical signs, body condition and estimated herd size. Two or three animals per herd were randomly selected to enable sampling of as many herds as possible.

In total 102 serum and corresponding probang samples were collected from buffalo; these comprised samples from MME (*n* = 40), TSE (*n =* 33) and ME (*n* = 29) with approximate animal ages ranging between 8 months and 19 years. The probang samples were diluted 1:1 in phosphate buffered saline (PBS) (pH 7.2) supplemented with 0.01% bovine serum albumin (BSA), 0.002% phenol red and 0.25% antibiotics (Pen-Strep-Neo, Sigma-Aldrich, St. Louis, MO, USA), stored in liquid nitrogen and transported to FMDL, Embakasi, where they were stored at −80°C.

### Samples from cattle

Taking into consideration the time of sampling, quality of material and the geographical source with a focus on districts around ecosystems of interest for this study and the sampling period (2008-2012), cattle epithelial samples were selected from the repository of all Kenyan field samples at the FMDL, Embakasi (Figure 1 and Table 1).

**Table 1:** **List of the 49 FMD viruses analysed in this study**

*Serial no.	**Sample reference no.	**Date of collection**	**Host species**	**District of origin**	**Province**	**Serotype on Ag ELISA**	**Ct value on RT-qPCR**	**Serotype on VP1 sequencing**	**Accession no.**
1	Ken/TSE1/2012	31/07/2012	Buffalo	Taita	Coast	SAT 1	17.19^a^	SAT 1	KP263443
2	K159/2012	19/12/2012	Cattle	Meru	Eastern	SAT 1	24.1^b^	SAT 1	KP263444
3	K127/2011	01/12/2011	Cattle	Taita-Taveta	Coast	SAT 1	9.96	SAT 1	KP263445
4	K56/2010	24/03/2010	Cattle	Laikipia	Rift Valley	SAT 1	24.48	SAT 1	KP263446
5	KenMMB37/2012	25/02/2012	Buffalo	Narok	Rift Valley	SAT 2	25.12	SAT 2	KP263447
6	K146/2012	31/10/2012	Cattle	Nakuru North	Rift Valley	SAT 2	14.66	SAT 2	KP263448
7	K125/2012	05/09/2012	Cattle	Kisii	Nyanza	SAT 2	14.6	SAT 2	KP263449
8	K126/2012	09/10/2012	Cattle	Nakuru	Rift Valley	SAT 2	15.64	SAT 2	KP263450
9	K53/2012	25/05/2012	Cattle	Kericho	Rift Valley	SAT 2	17.79	SAT 2	KP263451
10	K26/2012	21/02/2012	Cattle	Narok South	Rift Valley	SAT 2	10.44	SAT 2	KP263452
11	K28/2012	25/02/2012	Cattle	Nyandarua	Central	SAT 2	13.57	SAT 2	KP263453
12	K10/2012	20/01/2012	Cattle	Bomet	Rift Valley	SAT 2	23.11	SAT 2	KP263454
13	K46/2012	27/04/2012	Cattle	Nakuru North	Rift Valley	SAT 2	15.7	SAT 2	KP263455
14	K128/2011	01/12/2011	Cattle	Nyandarua	Central	SAT 2	19.37	SAT 2	KP263456
15	K30/2012	28/02/2012	Cattle	Subukia	Rift Valley	SAT 2	14.03	SAT 2	KP263457
16	K138/2012	05/10/2012	Cattle	Gilgil	Rift Valley	A	11.31	A	KJ440872
17	K143/2012	19/10/2012	Cattle	Naivasha	Rift Valley	A	24.13	A	KJ440873
18	K148/2012	13/11/2012	Cattle	Nakuru North	Rift Valley	A	19.29	A	KJ440874
19	K154/2012	03/12/2012	Cattle	Koibatek	Rift Valley	A	23.5	A	KJ440875
20	K3/2013	09/01/2013	Cattle	Thika East	Central	A	22.56	A	KJ440876
21	K63/2009	31/03/2009	Cattle	Narok South	Rift Valley	A	25.75	A	KJ440871
22	K73/2008	23/08/2008	Cattle	Loitokitok	Rift Valley	A	23.17	A	KJ440870
23	K33/2010	01/12/2010	Cattle	Ijara	North Eastern	O	25.75	O	KP765607
24	K10/2009	26/01/2009	Cattle	Machakos	Eastern	A	28.65	N/A	N/A
25	K14/2013	28/01/2013	Cattle	Sotik	Rift Valley	A	No Ct	N/A	N/A
26	K140/2012	12/10/2012	Cattle	Koibatek	Rift Valley	A	30.12	N/A	N/A
27	K144/2012	25/10/2012	Cattle	Sotik	Rift Valley	A	29.44	N/A	N/A
28	K151/2012	26/11/2012	Cattle	Subukia	Rift Valley	A	26.69	N/A	N/A
29	K152/2010	07/12/2010	Cattle	Transmara	Rift Valley	A	25.4	N/A	N/A
30	K160/2012	28/12/2012	Cattle	Rongai	Rift Valley	A	27.41	N/A	N/A
31	K18/2013	31/01/2013	Cattle	Rongai	Rift Valley	A	16.74	N/A	N/A
32	K2/2013	09/01/2013	Cattle	Mogotio	Rift Valley	A	16.43	N/A	N/A
33	K5/2013	10/01/2013	Cattle	Murang’a	Central	A	18.38	N/A	N/A
34	K64/2010	04/06/2010	Cattle	Narok	Rift Valley	A	36.78	N/A	N/A
35	K7/2013	16/01/2013	Cattle	Nakuru	Rift Valley	A	27.58	N/A	N/A
36	K88/2010	14/05/2010	Cattle	Githunguri	Central	A	31.86	N/A	N/A
37	K43/2011	19/03/2011	Cattle	Suba	Nyanza	SAT 1	29.12	N/A	N/A
38	K78/2011	26/08/2011	Cattle	Lamu West	Coast	SAT 1	28.21	N/A	N/A
39	K8/2011	01/07/2011	Cattle	Nyeri South	Central	SAT 1	23.64	N/A	N/A
40	K84/2012	07/03/2012	Cattle	Kathonzweni	Eastern	SAT 1	13.77	N/A	N/A
41	K113/2012	09/08/2012	Cattle	Mathioya	Central	SAT 2	25.69	N/A	N/A
42	K122/2011	23/11/2011	Cattle	Njoro	Rift Valley	SAT 2	25.65	N/A	N/A
43	K127/2012	12/09/2012	Cattle	Kisumu East	Nyanza	SAT 2	24.62	N/A	N/A
44	K33/2012	01/03/2012	Cattle	Njoro	Rift Valley	SAT 2	24.06	N/A	N/A
45	K39/2012	16/03/2012	Cattle	Kenyanya	Nyanza	SAT 2	28.08	N/A	N/A
46	K54/2012	31/05/2012	Cattle	Mashuru	Rift Valley	SAT 2	21.16	N/A	N/A
47	K55/2012	04/06/2012	Cattle	Koibatek	Rift Valley	SAT 2	28.86	N/A	N/A
48	K78/2012	28/06/2012	Cattle	Naivasha	Rift Valley	SAT 2	22.07	N/A	N/A
49	K98/2012	22/07/2012	Cattle	Nyandarua West	Central	SAT 2	9.9	N/A	N/A

*No. 1-15 correspond to the numbers in Figure 1 showing the geographic origin of the 15 SAT 1 and SAT 2 cattle and buffalo FMD viruses sequenced and compared in this study, while No. 1-23 indicate all the 23 FMD viruses that were successfully sequenced in the entire study.

N/A - Not applicable since the sequence was not determined.

**Sample reference number: the letter (K) indicates the first letter of the name of the country of origin (Kenya), followed by the serial number of the isolate and the year of sampling.

^a^Ct value for buffalo samples based on 3D RT-qPCR assay.

^b^Ct value for all cattle samples based on 5’UTR RT-qPCR assay.

### Testing strategy

This study compared evidence of infection by FMDV in buffalo and cattle using serological and virological assays as recommended by the OIE terrestrial manual [30]. All serological tests (except VNT for antibodies against SAT 3) were performed at FMDL, Embakasi, while all tests on epithelia from cattle and probang samples from buffalo, including sequencing were performed at the National Veterinary Institute, Lindholm, Denmark.

Buffalo sera were screened for antibodies against FMDV non-structural proteins (NSPs) as an indicator of prior infection with FMDV. Serotype-specific antibody titres were initially determined using liquid phase blocking ELISAs (LPBEs) (for antibodies against all FMDV serotypes except Asia 1). Thereafter (due to expected cross-reactivity among ELISAs [31-33]), VNTs, which exhibit lower level of cross-reactivity [31,32], were performed for neutralizing antibodies against six serotypes of FMDV (all except Asia 1).

All buffalo probang samples were tested using the 3D coding region-targeted real time RT-PCR (3D RT-qPCR) assay and virus isolation (VI) was attempted. Harvests of samples that induced cytopathic effect (CPE) in primary bovine thyroid (BTY) cells were tested in antigen ELISA (Ag-ELISA) and in 5’UTR-targeted real time RT-PCR (5’UTR RT-qPCR). Harvests positive in Ag-ELISA and with sufficient FMDV RNA to generate an amplicon corresponding to the VP1 coding sequence were characterized by sequencing. Cattle epithelia were tested directly in the Ag-ELISA and using the 5’UTR RT-qPCR assay and when amplicons could be generated directly were sequenced.

### Laboratory methods

#### Detection of antibodies against FMDV non-structural proteins (NSPs) in buffalo sera

All 102 buffalo sera were screened using the PrioCHECK® FMDV NS kit (Prionics AG, Switzerland) to detect antibodies against the NSPs of FMDV. The assay was performed according to the manufacturer’s instructions. Sera were tested using a 1:5 dilution and optical density (OD) measured at wavelength 450 nm (OD_450_ test sample). The results were expressed as Percentage Inhibition (PI) relative to the negative control (OD_450_ max) as follows: PI = 100 − [OD_450_ test sample/OD_450_ max)] × 100. Sera with PI <50 were considered negative and sera with PI ≥50% positive.

#### Assay for antibodies against FMDV using serotype-specific liquid phase blocking ELISA (LPBE) in buffalo sera

The LPBE assay was performed on all 102 buffalo serum samples using a commercial kit (BDSL, Scotland, UK), in accordance with the OIE manual [30]. The antigens used for this assay were: O_1_ Manisa, A_22_ IRQ 24/64, C PHI 7/84, SAT 1 (105), SAT 2 Eritrea, SAT 3 (309), and Asia 1 Shamir as contained in the kit. The sera were tested in two-fold dilution series from 1/32 to 1/256. The results were expressed as the reciprocal of the last positive dilution (the titre); samples with titres ≥90 were considered positive in accordance with instructions in the OIE manual.

#### Assay for neutralising antibodies against FMDV in buffalo sera

VNTs were performed to detect neutralizing antibodies against six serotypes (all except Asia 1) on all the 102 buffalo sera to confirm the LPBE results and to clarify possible cross-reactions as described in the OIE manual [30]. Briefly, quadruplicate two-fold dilution series of serum samples were incubated for 1 hr in flat-bottomed tissue culture grade microtitre plates with about 100 TCID_50_ of each of the Kenyan FMDV vaccine strains (O K77/78, A K5/80, C K267/67, SAT 1 T155/71, SAT 2 K52/84) and a Zimbabwean SAT 3 isolate (SAT 3 ZIM 4/81). The use of these old isolates was based on previous experience [23] and their satisfactory performance during the annual World Reference Laboratory (WRL) proficiency tests. Subsequently, a suspension of baby hamster kidney (BHK) cells was added to the samples followed by incubation for 2 days at 37°C. For SAT 3, incubation was in primary swine kidney (SK) cells for 3 days at 37°C. The controls included titration of a standard positive serum, cell control and a ten-fold titration of the virus suspension. The final end point titres were calculated as described previously [34] and titres ≥45 were considered positive, 16-44 doubtful and <16 negative [30].

#### Serological data recording and statistical analysis

Serological results were recorded and descriptive statistics calculated in MS Excel 2007 (Microsoft Corporation). Analyses of estimated prevalences and Confidence Intervals were performed using the Survey toolbox software [35].

#### Epithelial and probang sample processing and virus isolation (VI)

All epithelia and probang samples were thawed at room temperature and processed as recommended in the OIE manual [30]. Epithelial samples from cattle were ground in Eagles minimum essential media supplemented with protein hydrolysate, 2% fetal calf serum and antibiotics (2 million I.U. benzyl-penicillin, 1 g dihydrostreptomycin sulphate, 0.5 g neomycin sulphate, 1 g streptomycin and 8.5 μg amphotericin per litre) using sterile sand, mortar and pestle to make a 10% (w/v) suspension. These lysates were tested directly in the Ag-ELISA and used for RNA extraction, RT-qPCR and sequencing (see below). Probang suspensions from buffalo were inoculated onto BTY cells for 1 hour at 37°C followed by a change of media and continued incubation. The cultures were examined after 24 and 48 hours and harvested when CPE developed. The CPE negative samples were harvested by freeze-thawing and inoculated onto fresh cells for another 48 hours. CPE positive samples were harvested, while cultures negative after 2 passages were discarded. Positive harvests were tested in the Ag-ELISA and tested in the 5’UTR RT-qPCR assay (see below).

#### Detection of FMDV RNA using quantitative real time RT-PCR (RT-qPCR)

Quantitative RT-qPCR assays targeting the FMDV 3D coding sequence of the FMDV RNA were performed on buffalo probang samples as described previously [36] using a Superscript III/Platinum Taq one-step RT-qPCR kit (PE Biosystems, Life Technologies, Carlsbad, California, USA) with 3D probe (5′-FAM-TCCTTTGCACGCCGTGGGAC-TAMRA 3′), forward primer (5′-ACTGGTTTTACAAACCTGTGA-3′) and reverse primer (5′-GCGAGTCCTGCCACGGA-3′). In addition, RT-qPCR assays targeting the FMDV 5’ UTR were performed on all the 47 cattle epithelia and CPE positive buffalo harvests using TaqMan® Universal 2X PCR Master Mix (PE Biosystems, Life Technologies, Carlsbad, California, USA). The PCR was run, as described previously [37], using the FMDV MultiII IRES primers (FMDV Multi II forward primer and FMDV Multi II reverse primer) and FMDV Multi II-288 probe (FAM-labelled).

#### Antigen detection ELISA (Ag-ELISA)

Ag-ELISA to detect the presence of FMDV was performed as described in the OIE manual [30] and by [38], and, when positive, to determine the serotype. Samples with an OD difference between sample and negative control of >0.2 were considered positive, while those between 0.1 and 0.2 were considered inconclusive and repeated.

#### Sequencing of the FMDV VP1 coding region

Viral RNA was extracted from Ag-ELISA positive harvests of cell culture generated using buffalo probang samples and cattle epithelia samples. This was achieved by using the QIAmp® RNA blood mini kit (Qiagen, Hamburg, Germany) following the protocol for extraction of total RNA as described by the manufacturer. The RNA was eluted using 60 μl of RNase-free water and stored at -80°C. It was tested using the 5′UTR RT-qPCR assay. For selected RNAs the Ready-To-Go You-Prime First-Strand beads (GE Healthcare Life Sciences, Uppsala, Sweden) were used to synthesize new cDNA with random hexamer primers (pdN6).

The FMDV cDNA sequences were amplified using the reverse primer 1.0 PN 15 (NK-72) [39] and forward primers 1.0-U PN E (AKS-2) [40] or 13-KPN 100 or 13-KPN 101 (Table 2). The latter two primers were designed from the sequences of the Kenyan SAT 1 (K127/2011) and SAT 2 (K10/2012) cattle samples from this study, respectively. The PCRs were performed as described previously [41] and the products (≈840 bp) were analysed by electrophoresis on 1.5 % agarose gels (Seakem GTG agarose in 1 X TAE - low EDTA buffer) at 120 volts for 30 min. in parallel with a 1 kb DNA ladder GeneRuler® (Fermentas, Vilnius, Lithuania).

**Table 2:** **Primers used for amplification of FMDV VP1 cDNA in this study**

**Primer name**	**Sequence (5ˈ-3ˈ)**	**Isolate of origin**	**Reference**
1.0 PN 15 (NK-72) (reverse)	GAAGGGCCCAGGGTTGGACTC	Accession no. AJ 539141	Mason et al., 2003 [35].
13-KPN 100 (forward)	GGGTGGBBGTSTWMCAGRTSACMGACAC	K127/2011	This study
13-KPN 101(forward)	CACTGCTAYCAYKCNGARTGGGA	K10/2012	This study
1.0-U PN E (AKS-2) (forward)	TTAACTACCACTTCATGTACACXG	Accession no AY593849	Sangula et al., 2010 [36].

V = A,C,G; H = A,C,T; B = C,G,T; S = C,G; W = A,T; M = A,C; R = A,G; Y = C,T; K = G,T; N = Any; X = Inosine.

PCR products were purified using SigmaSpin® Sequencing Reaction Clean-Up Columns (Sigma-Aldrich, St. Louis, MO, USA) following the manufacturer’s instructions. Quantification of products and cycle sequencing were performed as previously described [41]. Cycle sequencing in both directions was achieved using the same forward and reverse primers as for the RT-PCRs.

#### Sequence assembly, alignment and analysis

The nucleotide sequences were assembled and edited using SeqMan Pro software (DNAstar, Inc., Madison, WI, USA). Serotype identification of the sequences was achieved by comparison with Genbank data using the Basic Local Alignment Search Tool (BLAST) [42].

The buffalo and cattle VP1 coding sequences generated in this study were compared to selected FMDV SAT 1 and SAT 2 sequences from Kenyan cattle determined in previous studies, from the WRLFMD [43] and from Genbank (see list in Additional file 1: Table S1 and Additional file 2: Table S2). Sequence alignment was achieved using MUSCLE [44] incorporated in MEGA software version 5.2 [45] and trimmed to 639 nucleotides encompassing the complete VP1 coding region of the viral RNA genome. Substitution models were also determined in MEGA5.2 as earlier described [41], briefly, Maximum Likelihood fits of 24 different nucleotide substitution models were estimated and Akaike Information Criterion (AIC) was used. Non-uniformity evolution rates were modelled using discrete gamma distribution (G) and the Tamura Nei substitution model with gamma distribution and invariable rates (I) (TN93 + G + I) was chosen [45]. The evolutionary history was inferred using the neighbor-joining method [45] and a bootstrap consensus tree estimated from 1000 replicates [46]. Percentage nucleotide differences among taxa in the data sets were calculated using MEGA5.2 [45] and genetic distances compared using the *P*-distance.

## Results

None of the 102 buffalo sampled in this study had clinical signs suggestive of FMDV infections during the sampling, while all the 47 cattle samples analysed were from animals with apparent clinical signs of FMD (data not shown).

### Antibodies against FMDV non-structural proteins (NSP) in buffalo sera

Out of 102 buffalo sera tested, 65 (64%) had antibodies against FMDV NSPs; these were distributed between the three different ecosystems as follows: MME 36/40 (90%: CI = 84–100%); ME 17/29 (59%: CI = 51–71%) and TSE 12/33 (36%: CI = 22–40%) (Table 3).

**Table 3:** **Detection of FMDV RNA and antibodies against FMDV in buffalo from selected wildlife ecosystems in Kenya**

			**No. of samples positive among the 3 ecosystems per test**										
					**LPBE per serotype (titres ≥90)**						^**d**^ **VNT per serotype**		
**Ecosystem**	**Total no. sampled**	**Sampling period in 2012**	**Real time 3D RT-qPCR**	**NS ELISA**	**O**	**A**	**C**	**SAT 1**	**SAT 2**	**SAT 3**	**SAT 1 only**	**SAT 2 only**	^**e**^ **SAT 1 & SAT 2**
^**a**^ **MME**	**40**	Feb.	22	36	16	10	5	15	34	12	11	18	8
^**b**^ **ME**	**29**	March	6	17	25	16	3	10	16	19	4	14	3
^**c**^ **TSE**	**33**	Aug.	15	12	29	27	11	32	24	19	29	21	19
**Total**	**102**		**43**	**65**	**70**	**53**	**19**	**57**	**74**	**50**	**44**	**53**	**30**

^a^Maasai-Mara Ecosystem; ^b^Meru Ecosystem; ^c^Tsavo Ecosystem; ^d^VNT assay did not detect any antibodies against FMDV serotypes O, A, C and SAT 3. ^e^Positive for both SAT 1 and SAT 2.

### Serotype-specific antibodies against FMDV in buffalo sera detected by LPBE

Generally, antibodies were detected by the LPBE against each of the six FMDV serotypes tested for (O, A, C, SAT 1, SAT 2 and SAT 3). Only three of the 102 buffalo samples were negative (with titres ≤90) for antibodies against all six serotypes tested for by LPBE, while 15, 15, 10, 19, 19 and 21 samples were positive for antibodies against all six, five, four, three, two and one serotype, respectively (data not shown). Moreover, high antibody titres (≥256) against serotypes O, A, SAT 1, SAT 2 and SAT 3 were found in 33, 20, 37, 39 and 29 sera, respectively (data not shown). The positive samples were distributed in the three ecosystems in different proportions as indicated in Table 3, i.e. SAT 2 dominating in MME, O/SAT 3 in ME and SAT 1/O in TSE.

### Detection of neutralising antibodies against FMDV by VNT in buffalo sera

The buffalo sera were also tested in VNT assays. In contrast to the LPBE results, there was no evidence for the presence of neutralising antibodies against FMDV serotypes C and SAT 3 among the 102 buffalo in the three ecosystems. Only one and two sera had neutralizing antibodies against serotypes O and A, respectively; moreover, these three sera had higher or equal titres against SAT 1 and/or SAT 2 and thus the apparent presence of anti-O and anti-A antibodies may result from cross reactivity in the assays. In contrast, neutralising antibodies against serotypes SAT 1 and SAT 2 were detected in 44/102 (43%) and 53/102 (52%) samples, respectively. Thirty of these samples were positive for antibodies against both serotypes and generally had higher titres against SAT 2 than against SAT 1, while 14 and 23 samples only had antibodies against SAT 1 or SAT 2, respectively (Table 3). Altogether 67 sera had neutralising antibodies against FMDV, including 41 of the 65 sera with antibodies against NSP, meaning that 24 and 26 sera only were positive in one of these test systems (data not shown). The distribution of the positive sera between the three ecosystems is shown in Table 3. MME and ME had higher levels of neutralising antibodies against SAT 2 than against SAT 1, while TSE predominantly had SAT 1 neutralising antibodies.

### Presence of FMDV and FMDV RNA in buffalo probang samples

Among the buffalo probang samples, 43/102 (42%) had evidence of FMDV RNA as detected by the 3D RT-qPCR assay on the original, non-passaged samples (Table 3) with Ct values ≤32 and were distributed as follows: MME (22/40), ME (6/29) and TSE (15/33). Twenty seven of these 43 (63%) positive samples came from buffalo with neutralising antibodies against FMDV SAT 1 and/or SAT 2.

The 43 buffalo probang samples that were positive in the 3D RT-qPCRs were inoculated onto BTY cells. Thirty three of these samples induced CPE, but only two of the cell harvests tested positive in the FMDV Ag-ELISA and were identified as SAT 1 and SAT 2, respectively (data not shown). Moreover, only these same two cell culture harvests (from MME and TSE) contained significant levels of FMDV RNA (Ct values of 17.19 and 25.12, Table 1) in the 5’UTR RT-qPCR assay and were used for VP1 sequencing following RT-PCR.

### Presence of FMDV antigen and RNA in cattle epithelia

All the 47 cattle epithelium samples (directly tested without virus isolation) were positive on Ag-ELISA, and their distribution between the serotypes was as follows: O (1); A (20); SAT 1 (7) and SAT 2 (19) (Table 1). All but two of the 47 cattle epithelial samples had Ct values <32 and amplicons corresponding to the VP1 coding sequence were successfully generated and sequenced from 21 of them (Table 1).

### Determination of VP1 coding region sequences

A total of 23 FMDV VP1 coding region sequences were successfully generated in this study (Table 1). These comprised two buffalo sequences (generated after VI) and 21 cattle sequences (directly sequenced from epithelial suspensions without VI). The buffalo sequences were identified as FMDV SAT 1 and SAT 2 originating from TSE and MME, respectively, while the 21 cattle sequences were identified as O (1), A (7), SAT 1 (3) or SAT 2 (10). The serotype identification, based on the sequence comparison (using BLAST), of samples from both species corresponded to the Ag-ELISA results. The cattle viruses originated from various parts of the country and for the purpose of this study, only the 3 serotype SAT 1 and the 10 SAT 2 cattle sequences were included in the phylogenetic analysis presented here, while the serotype O and A sequences were analysed elsewhere [41,47] (Table 1).

### Statistical analysis and interpretation of FMDV prevalences in buffalo

The overall prevalence of antibodies against FMDV in buffalo as determined from the NSP assays was 64%, and the detection of FMDV RNA in probang samples by 3D RT-qPCR was 42%. Thirty seven of the 43 (86%) animals with FMDV RNA in the pharynx also had antibodies against FMDV NSPs, while six buffalo had FMDV RNA in their pharynx without being antibody positive and 28 buffalo had antibodies against NSP without FMDV RNA in the pharynx (data not shown). The two positive buffalo isolates came from animals that had RNA in the pharynx, had antibodies against NSPs and were sero-positive (on VNT assay) for the same serotypes (SAT 1 and SAT 2) of virus.

### VP1 coding sequence analysis in this study

**Figure 2:**
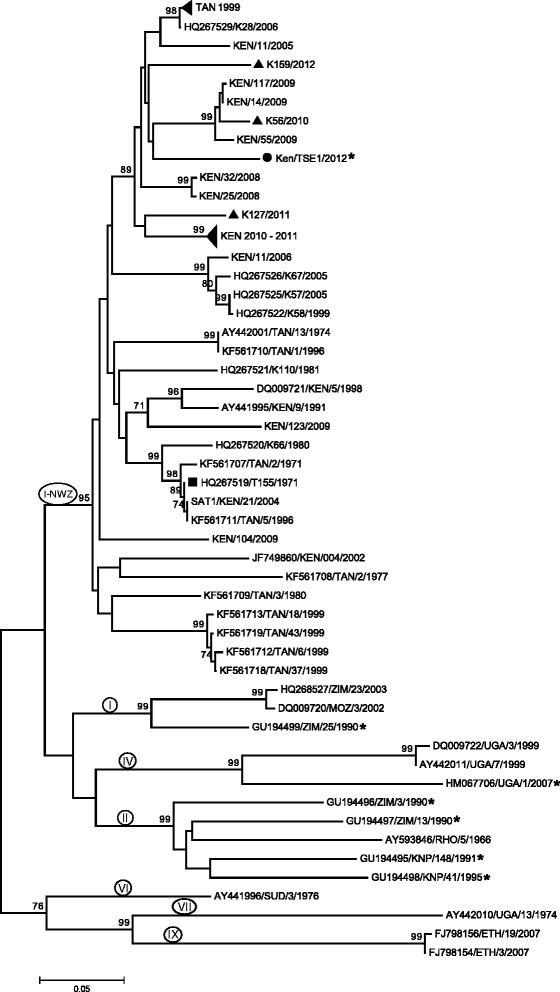
**Neighbor-Joining tree depicting one Kenyan foot-and-mouth disease virus (FMDV) SAT 1 buffalo sequence from this study (●) compared to recent SAT 1 Kenyan cattle sequences from this study (▲) and from World Reference Laboratory for FMD (WRLFMD) (◄); recent Ugandan buffalo sequence (UGA/1/2007); the current Kenyan vaccine strain (■); older Kenyan cattle sequences from WRLFMD (with prefix “KEN”) and Genbank; and selected cattle and buffalo sequences from eastern and southern Africa obtained from GenBank and listed in the Additional file** **1** **: Table S1.** Sequences from buffalo species are marked with an asterisk (*). Only Bootstrap test values above 70 are shown on the branches. Topotypes are indicated on the branches by the prefix I-NWZ for one topotype and by Roman numbers for the rest.

**Figure 3:**
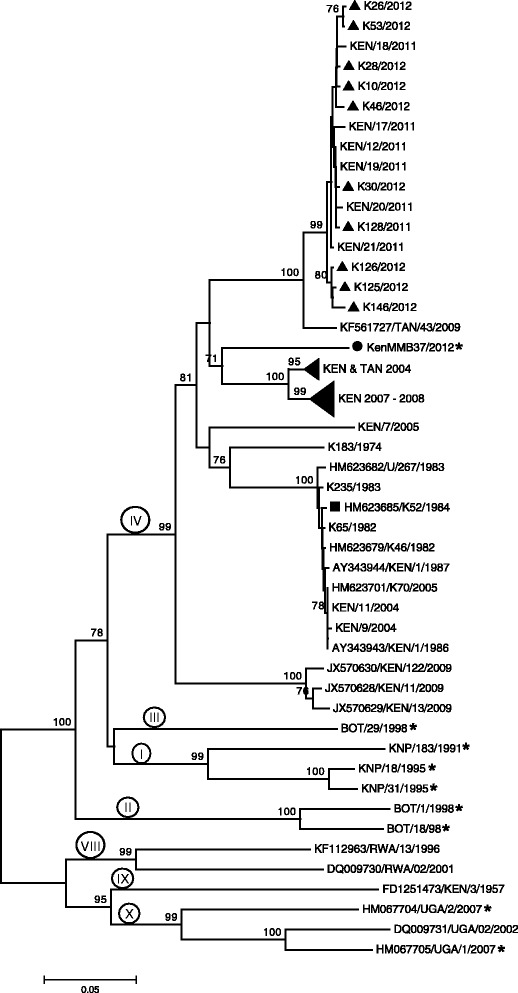
**Neighbor-Joining tree showing one Kenyan foot-and-mouth disease virus (FMDV) SAT 2 buffalo sequence from this study (●) compared to recent SAT 2 Kenyan cattle sequences obtained in this study (▲) and from World Reference Laboratory for FMD (WRLFMD) (With the prefix “KEN”); recent buffalo sequences from Uganda (UGA/1/2007 and UGA/2/2007); the current Kenyan vaccine strain (■); older Kenyan cattle sequences from WRLFMD (◄) and from Genbank; and cattle and buffalo sequences from eastern Africa and southern Africa obtained from GenBank and listed in the Additional file** **2** **: Table S2.** Sequences from buffalo species are marked with an asterisk (*). Only Bootstrap test values above 70 are shown on the branches. Topotypes are indicated by Roman numbers on the branches.

A total of 73 FMDV SAT 1 and 75 SAT 2 VP1 coding sequences (including the 15 SAT 1 and SAT 2 FMDV sequences generated in this study) were analysed in combination with sequences derived from other FMDVs originating in Kenya, other countries in eastern Africa and also other regions of Africa that were available from Genbank and WRLFMD (see Additional file 1: Table S1 and Additional file 2: Table S2). The estimated phylogenetic trees, using the Neighbor-Joining method, for the SAT 1 and SAT 2 virus sequences are shown in Figures 2 and 3 respectively.

The four SAT 1 VP1 coding sequences generated in this study comprised one (KenTSE1/2012) that was collected from a buffalo in TSE in Taita district, coast province, and three that were collected from cattle in different areas, namely Taita-Taveta (K127/2011), Laikipia in Rift Valley province (K56/2010) and Meru in Eastern Province (K159/2012) (Table 1, Figures 1 and 2). The 2012 buffalo isolate (KenTSE1/2012) clustered within the same topotype (I-NWZ) as both recent and older FMDV cattle isolates from Kenya but belonged to a separate, independently evolving lineage, from the cattle isolates (Figure 2). However, it shared a recent common ancestor with some recent Kenyan cattle viruses including K159/2012, K56/2010 and some of the 2009 group of viruses. Within the VP1 coding sequences, the buffalo KenTSE1/2012 isolate had 11%, 10% and 9% nt difference from these recent Kenyan cattle virus sequences, respectively. In addition, KenTSE1/2012 had 10% and 13% nt difference from the isolate found in cattle in the same district (Taita) (K127/2011) and from the current vaccine strain (T155/1971), respectively. This buffalo isolate also had >10% nt difference from the other cattle viruses collected in 2010-2011 from various districts in various regions of the country (data not shown). It is also noteworthy that these recent SAT 1 cattle sequences clustered within a separate lineage from the current vaccine strain. When compared to other African buffalo derived FMDV sequences, it was apparent that the KenTSE1/2012 belonged to a separate topotype (I-NWZ) (Figure 2). This isolate had 27% nt difference from the 2007 buffalo isolate from neighbouring Uganda (UGA/1/2007), 23% nt difference from the 1990 buffalo isolates from Zimbabwe (ZIM/3/1990 and ZIM/13/1990) and 24% nt difference from those collected in Kruger National Park (KNP/148/1991 and KNP/41/1995) in South Africa (data not shown).

The SAT 2 VP1 coding sequences included 11 sequences generated in this study (Table 1). These comprised 10 cattle sequences from various regions of the country and one buffalo sequence (KenMMB37/2012) from MME in Narok district of the Rift Valley province. The buffalo isolate grouped within the same topotype (IV) as some Tanzanian cattle viruses from 2004 and the Kenyan cattle viruses, including the viruses from 2004-2005 and 2007-2008, but has evolved as an independent lineage (Figure 3). Moreover, this Kenyan buffalo isolate also belonged to a different lineage from the current vaccine strain (SAT 2 K52/1984) (Figure 3) with 14% nt difference (data not shown) in this part of the genome. Comparisons of this buffalo isolate with the recent 2011-2012 cattle viruses showed >13% nt difference. It was also notable that, these recent SAT 2 cattle isolates clustered within a separate lineage (within topotype IV) than the current vaccine strain (Figure 3). Compared to the other buffalo sequences in eastern Africa, KenMMB37/2012 belonged to a different topotype (IV) and had on average >21% nt difference from the recent buffalo SAT 2 Ugandan viruses (UGA/1/2007 and UGA/2/2007) that grouped within East Africa topotype X (Figure 3). Similarly, this Kenyan buffalo SAT 2 virus belonged to a different topotype from the 1998 viruses from Botswana and the 1991-1995 South African (KNP) group of viruses and had on average, 20-30% nt differences from these viruses (data not shown).

## Discussion

This study has characterised and compared FMDVs that have recently infected buffalo and cattle in Kenya using a combination of assays. The PrioCHECK® FMDV NS ELISA demonstrated an overall seroprevalence of antibodies against FMDV NSPs of 64% in the studied Kenyan buffalo populations, which is comparable to the 68 % recorded by [25] in buffalo in eastern Africa but lower than the 74% and 85% reported in Ugandan buffalo in 2001-2003 and 2005-2008, respectively [15,27]. For comparison, the seroprevalence in Kenyan cattle in 2010 was 52.5% [48]. The NSP antibody seroprevalence varied between the three investigated ecosystems, with the highest recorded in MME followed by ME and lastly TSE; this is comparable to the reported variation in buffalo NSP antibody seroprevalence between Ugandan national parks [15,27].

Serotype-specific LPBE identified high titres (≥90) of antibodies against each of the six FMDV serotypes tested for, however, essentially only the antibodies against FMDV SAT 1 and SAT 2 were confirmed by VNT, suggesting high levels of cross-reactions in the commercial LPBE assay that was used. Such cross-reactions have been experienced using other serotype-specific antibody ELISAs in buffalo populations in Uganda [15,27] and in eastern Africa [25], as well as with sera from FMDV infected cattle with fresh or healing lesions (1-14 days after infection) [41,49]. However, clearer results have been obtained using SPBE in domestic ruminants sampled 2-4 months after infection [38,49] and in pigs sampled during an outbreak of SAT 1 in Kenya [23]. Moreover, since individual buffalo and buffalo herds are known to carry and maintain FMDV infections for a long time [11], it is likely that they are continuously exposed to FMDVs, resulting in the existence of animals with multiple previous infections by the virus. Therefore, high levels of cross-reactivity are to be expected due to boosting of antibodies against shared epitopes between the serotypes [50]. Consequently, the serotype-specific ELISAs may not be expected to give clear results in free-ranging African buffalo, underpinning the necessity of collecting and sequencing the circulating FMDVs from this species [26,41].

It is noteworthy that none of the sampled buffalo in this study had clinical signs suggestive of FMD, despite the fact that 42% (based on Ct ≤32) of the probang samples were positive for FMDV RNA by RT-qPCR. Furthermore, SAT 1 and SAT 2 FMDVs were each isolated from buffalo probang samples. This is not surprising because infection in African buffalo with FMDV has been known to be largely sub-clinical [8]; thus our results concur with previous studies in the region [15,27] and with three experimental infection studies in buffalo [51-53].

In this study, we isolated and characterised two FMD viruses, one SAT 1 and one SAT 2, from buffalo probang samples, while serotypes O (1), A (7), SAT 1 (3) and SAT 2 (10) FMDVs, respectively, were characterised directly from epithelial samples from acutely infected cattle from different regions of Kenya. These findings agree with previous reports that have found these four serotypes in circulation in Kenyan cattle [4] and confirm the continued presence of multiple serotypes of FMDV in Kenya since FMD was first diagnosed in 1932 [43]. In addition, the study also found that the recent SAT 1 and SAT 2 FMDVs in cattle and buffalo were divergent from the current vaccine strains, consistent with findings for serotypes O and A [41,47]. This finding raises concerns regarding the effectiveness of currently available vaccine strains against circulating viruses, suggesting the need for vaccine matching.

Among the SAT serotypes, the occurrence of SAT 3 FMDV has been mainly associated with buffalo and neutralising antibodies against SAT 3 have previously been demonstrated (albeit in lower proportions than the other SATs) in buffalo populations in Kenya [25], while in neighbouring Uganda both antibodies [15,26,27] and virus [26] have been reported in buffalo. Furthermore, the presence of SAT 3 virus in a long horned Ankole calf in Uganda has recently been reported for the first time [5]. Interestingly, the current study did not find SAT 3 FMDV or neutralising antibodies against SAT 3 (by VNT), neither among cattle nor within the buffalo populations in the three ecosystems studied within Kenya. These findings are consistent with reports from the WRLFMD [43] and records at the FMDL, Embakasi, that FMDV SAT 3 has never been detected in Kenya. Similarly, there was no evidence for the presence of serotype C in either buffalo or cattle. This serotype has not been isolated in Kenya since 2004 [54], although some serological evidence (subject to the caveats about antibody ELISA cross-reactivity) for exposure to this serotype has been found recently among cattle in Kenya [55], buffalo in neighbouring Uganda [27] and regionally among cattle in Eritrea [56] and Ethiopia [57]. There is a need for wider and more comprehensive studies among all FMDV susceptible species to verify the absence of these two serotypes in the country, considering the widespread movement of livestock and wildlife within and across national borders.

FMDV isolation, antigen detection ELISA and sequencing have been commonly performed on cattle samples in Kenya but rarely in other susceptible species (wildlife included) as is evident from the records of the FMDL, Embakasi, the WRLFMD [43] and from a recent study [23]. In the present study, the antigen ELISA and sequencing were fully consistent with each other. Taken together with the serological data, this study provided evidence that FMDV serotypes SAT 1 and SAT 2 are in circulation within the three buffalo ecosystems studied. This finding is consistent with an earlier field survey in 1979 among buffalo in MME and cattle around this ecosystem which isolated FMDV SAT 1 and SAT 2 from buffalo [24].

Guided by the principles published for SAT viruses that nt differences >20% define separate topotypes [58], this study found that although the SAT 1 buffalo virus identified in this study clustered within the same topotype (I-NWZ) as both recent and older FMDV isolates from Kenya, it belonged to a separate independently evolving lineage. Interestingly, even one virus (K127/2011) from cattle in the same district (Taita-Taveta) as the SAT 1 buffalo isolate did not group within the same lineage. However, the phylogenetic tree also showed that although this buffalo virus has been evolving independently from the cattle viruses, they originated from a common ancestor (Figure 2), suggestive of an ancestral species jump [10]. A similar trend was observed with the SAT 2 buffalo isolate KenMMB37/2012 collected from MME that belonged to the same topotype (IV) as both recent and earlier SAT 2 Kenyan cattle viruses but had ≥13% nt difference from them, including a recent virus from Narok district (K126/2012) in which MME is located (Figure 3).

These findings (albeit with a very small number of buffalo viruses) are consistent with the idea that there may be largely independent cycles of FMDV circulations in Kenya; one that occurs within buffalo populations and another within livestock populations, concurring with earlier observations indicating that eastern Africa may be experiencing these separate FMDV cycles [59]. This idea is also consistent with the absence of evidence from the current study of other serotypes circulating among buffalo populations either from virological or serological assays, yet serotypes O and A were frequently found in cattle.

The findings in this study contribute to the debate of whether FMD is mainly a disease of livestock in endemic Africa [17]. On the one hand, natural transmission from buffalo to livestock has been demonstrated in Zimbabwe [60,61] and South Africa [9], on the other hand, the real risks posed by carrier animals to susceptible hosts have not been adequately quantified [62]. However, the results presented here indicate that although other serotypes (O and A), together with SAT 1 and SAT 2 FMDVs are constantly circulating in Kenyan cattle, buffalo mainly harbour SAT serotypes. The current Kenyan findings are in agreement with previous studies in eastern Africa [24,25,27] and consistent with wildlife playing a limited role in the epidemiology of FMD in cattle in eastern Africa. However, owing to the limited number of buffalo viruses in our data set, it is not possible to draw firm conclusions.

## Conclusions

This study found that four serotypes of FMDV (O, A, SAT 1 and SAT 2 circulate among cattle in different regions of Kenya. Buffalo species were found to harbour SAT 1 and SAT 2 serotypes with no virological or serological evidence for other serotypes. Moreover, there was no evidence for the recent occurrence of serotypes C and SAT 3 in cattle or buffalo. The identified African buffalo virus lineages in the wild have apparently evolved separately from lineages found in livestock in Kenya and in the region. Hence, FMD control in Kenya should primarily focus on reducing the high virus burden among livestock and subsequently limit the association of livestock with wildlife. However, due to the limited data in this study, there is need for more comprehensive research incorporating a larger number of buffalo viruses and deeper analysis including evolutionary tracking of the origins of the viruses to determine the role of buffalo in the epidemiology of this disease.

## Additional files

Additional file 1: Table S1. List of 73 foot-and-mouth disease virus (FMDV) SAT 1 sequences included in the phylogenetic tree in Figure 2 of this study. Additional file 2: Table S2. List of 75 foot-and-mouth disease viruse (FMDV) SAT 2 sequences included in the phylogenetic tree in Figure 3 of this study.
